# Correlates of Delayed Initial Contact to Emergency Services among Patients with Suspected ST-Elevation Myocardial Infarction

**DOI:** 10.1155/2021/8483817

**Published:** 2021-09-14

**Authors:** Osayi Lawani, Nicholas Gorman, Fiona Gorman, Jiries Ganim, Stefano Sdringola-Maranga

**Affiliations:** ^1^HCA Houston Healthcare Kingwood, 22999 US Hwy 59 N, Kingwood, TX 77339, USA; ^2^Keck Graduate Institute, 535 Wharton Dr, Claremont, CA 91711, USA; ^3^California State University Long Beach, 1250 Bellflower Blvd., Long Beach, CA 90840, USA; ^4^The University of Texas Health Science Center at Houston, 7000 Fannin St, Houston, TX 77030, USA; ^5^Vital Heart and Vein, 18450 Hwy 59, Humble, TX 77338, USA

## Abstract

**Background:**

Early diagnosis and treatment of a patient displaying symptoms of myocardial ischemia is paramount in preventing detrimental tissue damage, arrhythmias, and death. Patient-related hospital delay is the greatest considerable cause of total delay in treatment for acute myocardial infarction.

**Objective:**

To identify patient characteristics contributing to prehospital delay and ultimately developing health interventions to prevent future delay and improve health outcomes.

**Methods:**

A retrospective chart review of 287 patients diagnosed with ST-elevation myocardial infarction (STEMI) was evaluated to examine correlates of patient-related delays to care.

**Results:**

Stepwise logistic regression modeling with forward selection (likelihood ratio) was performed to identify predictors of first medical contact (FMC) within 120 minutes of symptom onset and door-to-balloon (DTB) time within 90 minutes. Distance from the hospital, being unmarried, self-medicating, disability, and hemodynamic stability emerged as variables that were found to be predictive of FMC within the first 120 minutes after symptom onset. Similarly, patient characteristics of gender and disability and having an initial nondiagnostic electrocardiogram emerged as significant predictors of DTB within 90 minutes.

**Conclusions:**

Individual attention to high-risk patients and public education campaigns using printed materials, public lectures, and entertainment mediums are likely needed to disseminate information to improve prevention strategies. Future research should focus on identifying the strengths of prehospital predictors and finding other variables that can be established as forecasters of delay. Interventions to enhance survival in acute STEMI should continue as to provide substantial advances in overall health outcomes.

## 1. Introduction

**Figure 1:**
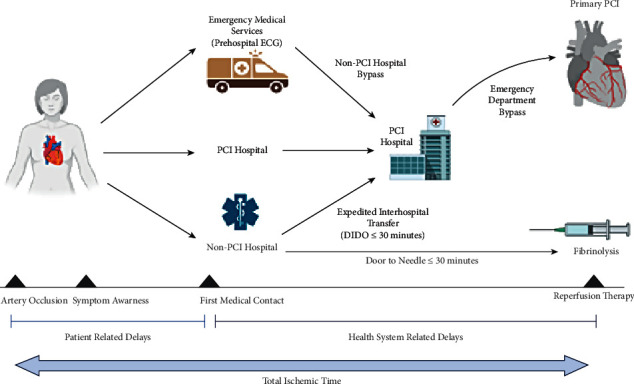
Illustration of the progression of care and treatment of myocardial infarction following initial consistent acute symptoms. Instances where patient-related delay and health system-related delay may occur are noted within the total ischemic time. DIDO = door-in-door-out; ECG = electrocardiogram; EMS = emergency medical services; PCI = percutaneous coronary intervention [4].

Heart disease is currently the most common cause of death in the United States and in most developed countries worldwide [1, 2]. Early diagnosis and treatment of a patient displaying symptoms of myocardial ischemia is paramount in preventing detrimental tissue damage, arrhythmias, and death [3]. The progression of care of a patient with acute chest pain from the time of consistent symptoms to the period of prehospital and then in-hospital treatment is known as the total ischemic time and ends with percutaneous coronary intervention (PCI) to establish coronary reperfusion via balloon angioplasty (Figure 1) [4, 5]. As indicated by the European Society of Cardiology guidelines on STEMI, PCI should be initiated within 60–90 minutes after the patient makes initial first medical contact (FMC) preferably to emergency medical services (EMS) or to the door of a PCI capable hospital, a metric denoted as door-to-balloon (DTB) time [6, 7] (Table 1) [8]. Guidelines from the American Heart Association designated a timeline of 120 minutes for non-PCI hospitals and 90 minutes for PCI capable facilities [9].

A considerable amount of time is lost during the initial phase of total ischemic time where patients are the main determinant of making FMC [10]. Patient-related delay is defined as the period a patient spends making the decision to contact EMS following the start of consistent acute symptoms, which is dependent on a patient's cognitive ability and overall clinical understanding of warning signs of myocardial infarction (MI) [11]. Identifying factors affecting prehospital delay would assist health professionals to design preventative interventions that target the high-risk population for patient-related delay and potentially decrease the prevalence of poor outcomes following the onset of a cardiac event. The purpose of this study was to perform an assessment of the possible contributing factors that may cause patient-related delay in initiating preliminary medical contact in the treatment of acute STEMI.

## 2. Methods

### 2.1. Study Design and Setting

All patients that presented to a single area hospital located in the Northeast region of Houston, Texas, with symptoms that led to acute MI between May 1, 2018, and May 1, 2020, were initially evaluated. From these patients, those who presented to the emergency department by any mode of transportation with a final diagnosis of acute STEMI and resulting activation of the catheterization lab for emergency treatment were considered for the study. Upon contacting EMS, services were dispatched to the location of the incident and the patient was evaluated. If there was suspicion of acute MI, a 12-lead electrocardiogram (ECG) was performed. If the ECG was interpreted as a STEMI or if there was high suspicion, EMS would then triage patients and contact the nearest emergent PCI treatment center to speak with the emergency department physician who also must agree with the probable high suspicion of acute STEMI. The physician then will contact the on-call interventional cardiologist to notify them of the impending patient. If the cardiologist agrees, the catheterization laboratory team is then activated. On arrival, patients were taken immediately to the catheterization laboratory for treatment. Of note, another acute PCI center was located within five miles from the facility in this study, which may or may not have affected the number of potential patients allocated to this hospital.

### 2.2. Participants

A total of 311 patients met the initial criteria for screening. Inclusion criteria included all patients who were diagnosed with STEMI and were in transit, in some capacity, to this designated PCI treatment center, regardless of age. Patients transferred from a free-standing emergency room, an outside clinic, or were visiting from out of town were also included within this study, as well as patients that suffered cardiac arrest or cardiogenic shock and were able to be resuscitated and undergo PCI. Excluded individuals consisted of patients that, after further investigation, were found to have experienced unstable angina, non-STEMI, and STEMI if it occurred while a patient was hospitalized at the time of symptom onset. Patients who also experienced cardiac arrest or hemodynamic instability that hindered immediate treatment with emergent PCI were also excluded. Of the initial 311 subjects, a total of 287 patients were ultimately selected and were evaluated for the study.

### 2.3. Data Gathering

Data were collected and retrospectively reviewed from electronic medical records that had available information at the time of admission in the emergency department and from the initial cardiologist consultation or critical care admission note. These data were provided by either the patient, family members, EMS, or prior records. Evaluation of the history and physical was used to screen for signs or symptoms of cardiopulmonary distress and the patient's noted time of consistent symptoms before seeking FMC. FMC was observed as the time that the patient contacted EMS or personally arrived at the hospital or at a clinic, although prior studies used time of first ECG taken as the approximated time of FMC [12]. The patient's demographics, time of arrival to the hospital, and DTB time were also analyzed, as well as laboratory results and the ECG performed. This information was either gathered or completed by a medical doctor, physician assistant, nurse practitioner, nursing staff, or patient registration representative.

In total, 21 potential independent variables of delay were selected from six main categories: demographic characteristics, socioeconomic status, clinical data, comorbidities, and prehospital and in-hospital factors (Tables 2 and 3). The categories and their variables were developed after an extensive literature review of overt clinical experiences and observations in medicine of what was hypothesized to cause patient-related delay.

Demographic characteristics included age, race, gender, marital status, residence, and distance of travel from the hospital. Socioeconomic status was evaluated by income level and insurance status. Education information could not be obtained as this variable was not ascertained at the time of admission or at any time during the patient's hospitalization. Clinical data involved notation of chest pain that began >24 hours before contacting EMS, hemodynamic stability before or on arrival to the emergency department, and diagnostic value of the initial ECG. Use of over-the-counter or prescription analgesics such as acetaminophen, aspirin, or nonsteroidal anti-inflammatory drugs (ibuprofen or naproxen) and illicit drug use were designated as “self-medicating” prior to arrival, as initial use of these medications would hinder the feeling of acute MI symptoms and the sense of urgency associated with it. This excluded any use of nitroglycerin following the initiation of chest pain. Low-density lipoprotein and troponin levels were also collected for overall observational review for the current study population. Obesity (BMI ≥ 30 kg/m^2^), diabetes mellitus, prior history of coronary artery disease, and prior history of stroke and disability (any physical or mental limitations that hinder activities of daily living) were variables collected under the grouping for comorbidity. The prehospital category included mode of arrival and was subcategorized as a private vehicle, EMS (via ambulance or flight services), or transfer, indicating translocation from another facility for a higher level of care. Finally, the in-hospital category evaluated DTB time, if applicable, and was further subdivided by ≤120 minutes and >120 minutes.

The dependent variable was designated as the duration of consistent symptoms before FMC. The primary outcome was the evaluation of patient-related delay >2 hours from the period of symptom onset to the patient's initial presentation to the emergency department. This was assessed by the preliminary history and physical documentation of either the treating emergency medicine team or by the cardiologist or intensivist who was consulted. The intervals of FMC time evaluated were <30 minutes, between 30 and 120 minutes, and >120 minutes. The time of >2 hours was designated as the cutoff due to this time frame being found in other studies as the greatest considerable cause of morbidity and mortality, as the maximum benefit for coronary reperfusion would be within the first two hours after symptom initiation [13]. The institutional review board of this facility approved the study protocol.

### 2.4. Statistical Analysis

Data were initially entered in excel (Excel 2016) and analyzed using IBM SPSS Statistics for Mac, version 26.0 (IBM Corp, Armonk, NY, USA). Univariate analyses were used to document patient demography and medical characteristics. The multivariate analysis consisted of stepwise, binary logistic regression modeling with forward selection (likelihood ratio) to examine which patient characteristics predicted FMC and DTB times. A *p* value of 0.05 was set for the level of statistical significance for all inferential analyses.

## 3. Results

Data were extracted from a total of 287 patients. Of these individuals, 162 people activated EMS as their FMC, whereas 71 people drove themselves to the emergency department, and 54 were transferred from an outside clinic or hospital for acute PCI. The demographic characteristics of the sample are summarized in Table 2. Of note, the sample was predominantly male and white, with most patients living in urban settings. Only approximately 1-in-10 MI patients were uninsured at the time of the study.

The medical characteristics of the samples are summarized in Table 3. Several risk factors for MI were common in the sample, including frequent overweight/obese status, diabetes, and high low-density lipoprotein levels. Very few patients reported having taken medication for their symptoms prior to arrival.

In order to determine which variables predict FMC ≥ 120 minutes of the onset of symptoms, stepwise logistic regression modeling with forwarding selection (likelihood ratio) was utilized (*n* = 273). The overall model was statistically significant (*χ*^2^ (5) = 40.02, *p* < 0.001) and accounted for between 13 and 20% of the variability observed in time to first EMS contact (Cox and Snell *R*^2^ = 0.13; Nagelkerke *R*^2^ = 0.20). Five predictors emerged as statistically significant, with living more than 10 miles from the hospital, being unmarried, self-medicating, patient disability, and hemodynamic stability all increasing the odds of taking >120 minutes to make FMC (see Table 4).

To determine which variables predicted whether a patient's DTB times were within 90 minutes, stepwise logistic regression modeling with forward selection (likelihood ratio) was utilized (*n* = 268). The overall model was statistically significant (*χ*^2^ (1) = 20.08, *p* < 0.001) and accounted for 7–13% of the variability observed in DTB times (Cox and Snell *R*^2^ = 0.07; Nagelkerke *R*^2^ = 0.13). Three predictors emerged as statistically significant, with male gender, patient disability, and nondiagnostic ECG all increasing the odds of DTB times of >90 minutes (see Table 5).

## 4. Discussion

The current study aimed to perform an assessment of contributing factors that may cause patient-related delay in initiating preliminary medical contact to identify high-risk STEMI patients and prevent adverse outcomes. While results from a recent study by Stehli et al. noted that self-transportation and female gender were markedly associated with a delay in FMC, results from our current study indicated that distance from the hospital, marital status, self-medicating, disability, and hemodynamic status were predictors for having patient-related delay of >2 hours, regardless of gender [11].

### 4.1. Distance

Our study found that patients who lived >10 miles from the hospital were 2.39 times more likely to delay FMC. This finding was consistent with previous literature. One study in India noted that longer distance, rural residence, and issues with transportation were found to be predictors for late presentation [14]. This same study also noted that this variable may not be generalizable to other populations as all areas and locations differ in health-seeking behavior and sociodemographic factors [14]. Another study found that although some individuals lived farther from the hospital, they only used ambulance services about 5% of the time, which may have indicated the lack of understanding of the importance of early EMS contact and the sense of urgency that is warranted with acute treatment [15]. Alternatively, the burden of the cost of using EMS could have been an issue. In countries like Singapore, ambulance services are free of charge due to public funding [9]. It was found that patients who utilized EMS were independently associated with shorter symptom-to-door times [16]. Increased public health, as well as individual education emphasizing the importance of seeking out EMS urgently following symptoms of acute MI, is likely needed to assist in reducing the incidence of delay in relation to distance or transportation as a barrier to obtaining acute medical care [9].

### 4.2. Marital Status

We found that patient-related delay can differ in relation to marital status. Individuals in our study were discovered to prolong making FMC if they were single. We noted that unmarried patients were 2.22 times more likely to delay seeking medical attention. A study in Sweden observed that both unmarried men and women had an increased case-fatality rate on the first day of experiencing chest pain related to acute coronary syndrome [17]. Other study results vary with these findings; however, being married could be attributed to having a better quality of life and overall lifestyle [1]. Khafaji et al. found that the absence of social support with accompanying psychological stress is associated with a delayed FMC, especially if someone was widowed [17]. There may be a difference in delay due to cultural norms that include close knit families where three generations of family members or relatives were all residing within the same home, as there is an increased level of support [17]. Furthermore, certain individuals of an older age may prefer for a family member to contact EMS when experiencing acute chest pain as it provides a degree of comfort [18]. As this variable has likely gone unnoticed for a cause of delay, it indicates that there is a need for inquiry of current home life to evaluate the specific needs of acute coronary syndrome patients. Individuals who live alone over the age of 50, especially with significant risk factors for a cardiac event, should be provided increased education and social support when planning disease management to decrease a potential delay in FMC.

### 4.3. Self-Medication

Medicating for discomfort following the initiation of symptoms related to acute MI was a significant cause of patient-related delay. We found that patients who self-medicated were 9.02 times more likely to delay FMC. Common medications include over-the-counter analgesics or another substance that may decrease the sensation of pain like narcotics, alcohol, illicit drugs, or herbal remedies. Self-distraction may also be used. Gartner et al. noted that, for individuals that self-medicated, there was a threefold increase in relative risk of prolonging delay with attempting to suppress these warning signs [18]. Another study found that, following multivariate analysis, several variables including opium abuse were associated with an increase in symptom-to-door time [15]. Furthermore, opiate use can contribute to recall bias when determining the time and duration of symptom initiation [12]. Masking symptoms hinder further symptom recognition and decrease the indication that urgent management is needed.

### 4.4. Disability

Prehospital disability describes the impairment of an individual, with or without comorbidities, who requires support in activities of daily living, which include dressing, transferring, bathing, or walking short distances [19]. We found that patients that had one or more disabilities were 4.38 times more likely to delay making FMC. Conversely, a previous study stated that prehospital disability did not significantly contribute to their multivariable model. This study, however, may have been underpowered to detect an effect on prehospital delay as most of the study participants were fully functional [19]. Comorbidities can be associated with disability and are attributed to causing a slew of somatic symptoms that a person experiencing signs of an acute MI may credit to a prior medical issue and may discredit as an atypical symptom of a serious cardiac event. One study noted that individuals who are experiencing a STEMI with few to no comorbidities were more likely to seek help sooner [20]. Delay related to this patient population is likely due to limited access to medical care from physical disability and possibly from being unable to appreciate the symptoms of an acute MI [21].

### 4.5. Hemodynamic Status

Hemodynamic instability was found to be a protective factor in total ischemic time. Patients who are unstable were less likely to delay making FMC. This finding is probably due to instability leading to symptoms that are found to be extremely distressing to the patient, family member, or a bystander who is witness to the decline in status. Extreme examples of hemodynamic instability are cardiogenic shock or cardiac arrest, both of which would prompt immediate contact of EMS. A previous study noted that patients suffering from cardiogenic shock had shorter symptom-to-door times in comparison to noncardiogenic shock patients [12]. Individuals with left circumflex artery occlusion showed longer symptom-to-door delay (average 190 minutes), whereas patients with left anterior descending artery occlusion had shorter symptom-to-PCI delay (average 170 minutes) [12]. Patients experiencing cardiogenic shock following MI arrive sooner than the general population, which is likely related to the feeling of impending doom that these individuals are experiencing initially during an acute MI [18]. Scholz et al. commented on the importance of prompt reperfusion of the coronary arteries with PCI being a major predictor of survival in a patient experiencing acute MI with accompanying cardiogenic shock [3].

Other important findings of the present study are that gender and disability emerged as predictors for delayed DTB time. Previous multivariate analysis showed that patients with longer symptom-to-door times influenced delay in DTB time and resulted in a higher probability of complications and overall mortality [16].

### 4.6. Female Patients

We found that female patients were less likely to have DTB time >90 minutes. Although women had a prolonged symptom-to-door time, their DTB time was decreased in comparison to males. In a prior Australian study, women were noted to have a higher rate of delay in initial presentation after symptom onset and increase delay in DTB time, as well as higher 30-day mortality [21]. It was also observed that, in studies conducted in Sweden, England, and Wales, women experiencing acute MI underwent less timely PCI and revascularization compared to men [21]. Many factors could have led to these prior findings. Women experiencing acute MI usually display associated atypical symptoms such as palpitations, cough, fatigue, headache, pain in the neck, jaw, and in between the shoulder blades, shortness of breath, and nausea. It is believed that providers have difficulty identifying these symptoms as prodromal indicators of an MI in relation to typical symptoms in men [20]. Health professionals may also underestimate the overall cardiovascular risk for myocardial ischemia in female patients [22].

The data presented in this study may differ from previous information for several reasons. Women in the United States and particularly in the region of this study may have a different outlook on cardiovascular health and treatment in comparison with other countries. With likely strict health agency regulation and increased physician reimbursement on adhering to current evidence-based guidelines on acute MI treatment, women may receive more consideration of their cardiovascular risk, obtain more invasive diagnostic medical testing and PCI, and may be provided more guideline-directed pharmacological medical therapy [22]. Providing equality in cardiovascular care and treatment and adhering to standardized STEMI protocol and initial checklists can be and has been shown to reduce mortality significantly for the general US public. The US has also increased its efforts in disseminating information regarding cardiovascular health to the public, as well as to women specifically through national campaigns like the Million Hearts 2022 and WISEWOMAN national initiatives through the Centers of Disease Control and Prevention and the Heart Truth campaign headed by the National Heart, Lung, and Blood Institute.

There may also be increased use of sex-specific troponin analysis in the US as compared with other countries where more women were identified, diagnosed, and treated as they were determined to be at a higher risk for experiencing a cardiac event [23]. It was noted in a previous study that using sex-specific thresholds increased the identification of women with acute MI by fivefold [22]. Additionally, the decrease in DTB time could be attributed to an increase in efficient triage at the study facility or incidentally more obvious initial ECG changes. Stehli et al. noted that it is possible that implementing a focused driven protocol that involves adequate early triage, timely activation of the catheterization team, medical therapy that is guideline-directed, and use of the radial artery as opposed to the femoral artery benefited women more so than men when treating STEMI [20]. Overall, there is a need for a concerted and constant effort worldwide to improve awareness in women, as well as physicians, of the risk factors and the variable signs and symptoms of acute MI, with appropriate treatment and management.

### 4.7. Disability

We also found that patients with one or more disabilities were 3.41 times more likely to have a DTB time >90 minutes. Findings also highlighted patients who had one or more disabilities as a priority population for reducing the total ischemic time. This delay may be due to difficulties with managing the patient while they are being stabilized and awaiting PCI in the emergency department or the catheterization laboratory. It is important for hospitals to investigate and implement tailored strategies to reduce DTB time and improve the outcomes for this specific patient population that faces challenges to seeking care at multiple levels.

### 4.8. Study Limitations

Although our study had adequate power, the data were collected from STEMI patients who were mostly white males from a more rural, single center in Texas. The findings may not be generalizable to other regions or more diverse populations. Disability was a significant cause of delay; however, there was a limited number of participants within this study with this characteristic; thus, the results may have been skewed. Data obtained were observational from the standpoint of prior records, and consequently, the data were susceptible to certain biases, input error, investigator error, and confounding. There was a lack of prehospital time variables that may have been collected from EMS such as exact time of FMC, EMS arrival to the patient location, and time of departure from the location. Timing had to be estimated from EMS general description of the medical event, patient-provided information, and emergency department reports. The time from symptom onset to contact of EMS listed on hospital documentation may have had some degree of skepticism, as patients might have had some inaccuracies with the noted time frame. Possible psychological and cognitive factors related to prolonged symptom-to-door time could not be fully analyzed as there was no in-depth interview performed with these patients. Other studies on this subject may have incorporated questionnaires and in-person interviews to collect their data. Lastly, this study was primarily conducted in the setting of a private, for-profit healthcare system, thus mostly excluding patients that are dependent on public health care. Further studies should be performed in the public sector to broaden the results of this subject to the overall general population.

### 4.9. Implications in Clinical Practice

Our retrospective review and logistic regression analysis were used to identify patient characteristics that would contribute to delay following the onset of persistent symptoms of acute MI leading to STEMI. In terms of measures to prevent patient-related delay to first EMS contact, health interventions should target the following patient subgroups: patients who live >10 miles from the hospital, unmarried patients who live alone, patients who may self-medicate instead of making FMC, and patients that have ≥1 disability or comorbidities. Health education should focus on earlier recognition of symptoms of acute STEMI, risk of masking symptoms by self-medicating, potential long-term health consequences of delaying care, and available support services and resources that may assist patients to overcome barriers to seeking care.

### 4.10. Future Direction

Future research should focus on identifying the strengths of prehospital delay forecasters and finding other variables that can be established as positive predictors of delay. This information can then be used to develop a scoring system that serves as a clinical assessment tool to identify high-risk patients who would benefit from early intervention. Developing predictive algorithms for groups with well-defined features would be convenient to create individualized and valid prevention strategies, particularly with the use of risk scores [24]. Further studies examining the causes behind delaying FMC among patients with one or more disabilities are also warranted. It is important to investigate specific types of impairments, as patients with an overt physical disability would need vastly different support services compared to patients with other kinds of disabilities such as loss of vision or hearing. This will assist in informing the appropriate social and support services that are required to reduce FMC for this patient population.

## 5. Conclusion

**Figure 2:**
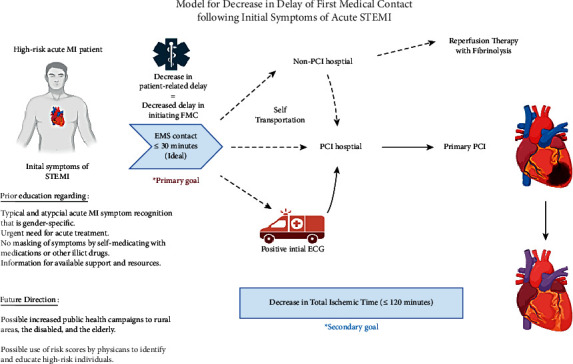
Central illustration: progression of care of myocardial infarction following initial consistent symptoms of acute MI. With consideration for prior patient education, public health campaigns, and the use of risk scores, a predicted decrease in patient-related delay will be noted with an overall reduction in prolonging FMC (≤30 minutes) and a decrease in total ischemic time (≤120 minutes). ECG = electrocardiogram; EMS = emergency medical services; FMC = first medical contact; MI = myocardial infarction; PCI = percutaneous coronary intervention; STEMI = ST-elevation myocardial infarction.

The span of delay between the beginning of symptom onset and contacting EMS has not changed since the 1980s [25]. To decrease the time in patient-related delay, patients who are at high risk for cardiovascular disease need to be able to recognize various symptoms of acute coronary syndrome other than chest pain which are in relation to STEMI and the critical need to seek out medical management. Strategies should continue to be developed to assist in shortening patient-related delay to provide substantial improvements in the survival of patients experiencing symptoms of acute MI (Figure 2).

## Figures and Tables

**Table 1:** Summary of time targets following acute MI from the European Society of Cardiology.

Intervals	Time targets
Maximum time from FMC to ECG and diagnosis	≤10 minutes
Maximum expected delay from STEMI diagnosis to primary PCI to choose PCI versus fibrinolysis (if this target time cannot be met, consider fibrinolysis)	≤120 minutes
Maximum time from STEMI diagnosis to wire crossing in patients presenting at primary PCI hospitals	≤60 minutes
Maximum time from STEMI diagnosis to wire crossing in transferred patients	≤90 minutes
Maximum time from STEMI diagnosis to bolus or infusion start of fibrinolysis in patients unable to meet primary PCI target times	≤10 minutes
Time delay from the start of fibrinolysis to evaluation of its efficacy (success or failure)	60–90 minutes
Time delay from the start of fibrinolysis to angiography (if fibrinolysis is successful)	2–24 hours

ECG = electrocardiogram; FMC = first medical contact; PCI = percutaneous coronary intervention; STEMI = ST-elevation myocardial infarction [8].

**Table 2:** Demographic characteristics of patients (*n* = 280–287).

Demographic characteristics	Mean (SD)	Freq (valid %)
Age	61.20 (11.94)	

*Gender*		
Male		206 (71.8)
Female		81 (28.2)

*Race/ethnicity*		
White (non-Hispanic)		209 (72.8)
Black		25 (8.7)
Hispanic		33 (11.5)
Others		20 (7.0)

*Residence*		
Rural, town, or village		78 (27.2)
Suburbs		55 (19.2)
Urban, city, or metropolitan		154 (53.7)

*Distance from hospital*		
<10 miles		141 (49.1)
>10 miles		146 (50.9)

*Income*		
<$45k		168 (58.5)
>$45k		119 (41.5)

*Marital status*		
Not married		166 (59.3)
Married		114 (40.7)

*Insurance*		
Uninsured		32 (11.1)
Government		155 (54.0)
Private		100 (34.8)

*k* = $1000. SD = standard deviation.

**Table 3:** Medical characteristics of patients (*n* = 267–287).

Demographic characteristics	Mean (SD)	Freq (valid %)
*BMI category*		
Underweight (≤18.5 kg/m^2^)		5 (1.7)
Normal (18.6–24.9 kg/m^2^)		49 (17.1)
Overweight (25–29.9 kg/m^2^)		121 (42.2)
Obese (30–39.9 kg/m^2^)		97 (33.8)
Morbidly obese (≥40 kg/m^2^)		15 (5.2)
*Arrival mode*		
Private vehicle		71 (24.7)
EMS		162 (56.4)
Transfer		54 (18.8)
Self-medicated prior to arrival (over-the-counter/prescribed analgesics or illicit drugs)		13 (4.5)
Prior out-patient cardiologist		112 (39.0)
Diabetes		99 (34.5)
Stroke history		20 (7.0)
Disability		27 (9.4)
Prior cardiac stent placement		43 (15.0)
Chest pain >24 hours prior to arrival		56 (19.5)
Known cardiac artery disease		113 (39.4)
*Hemodynamic stability upon arrival*		
Stable		226 (78.7)
Unstable		61 (21.3)
*LDL level*		
≤70 mg/dL		58 (21.7)
>71 mg/dL		209 (78.3)
*Highest troponin*		
≤0.40 ng/ml		52 (18.6)
>0.41 ng/ml		228 (81.4)

BMI = body mass index; EMS = emergency medical services; LDL = low-density lipoprotein.

**Table 4:** Multivariate, stepwise logistic regression of predictors of first EMS contact within 120 minutes (*n* = 273).

Independent variables	*B*	SE	Wald's *χ*^2^	d*f*	*p*	OR	95% CI
Distance (reference <10 miles)	0.87	0.31	7.71	1	0.005	2.39	(1.30–4.38)
Marital status: unmarried (reference: married)	0.80	0.31	6.81	1	0.009	2.22	(1.21–4.09)
Self-medicated	2.20	0.64	11.87	1	0.001	9.02	(2.57–31.64)
Disability	1.48	0.45	10.95	1	0.001	4.38	(1.82–10.61)
Hemodynamic instability	-0.92	0.43	4.48	1	0.03	0.40	(0.17–0.93)

Final model statistics: *χ*^2^ (5) = 40.02, *p* < 0.001, Cox and Snell *R*^2^ = 0.13, and Nagelkerke *R*^2^ = 0.20; EMS = emergency medical services.

**Table 5:** Multivariate, stepwise logistic regression of predictors of DTB time within 90 minutes (*n* = 268).

Independent variable	*B*	SE	Wald's *χ*^2^	d*f*	*p*	OR	95% CI
Gender (reference: male)	−1.01	0.52	3.78	1	0.05	0.36	(0.13–1.01)
Disability	1.23	0.50	5.93	1	0.02	3.41	(1.28–9.12)
Nondiagnostic ECG	2.53	0.44	11.88	1	0.001	6.62	(5.30–29.74)

Final model statistics: *χ*^2^ (5) = 20.08, *p* < 0.001, Cox and Snell *R*^2^ = 0.07, and Nagelkerke *R*^2^ = 0.13; DTB = door‐to‐balloon; ECG = electrocardiogram.

## Data Availability

The data are available upon request to the corresponding author.

## Abbreviations:

DTB: Door-to-balloon
ECG: Electrocardiogram
EMS: Emergency medical services
FMC: First medical contact
MI: Myocardial infarction
PCI: Percutaneous coronary intervention
STEMI: ST-elevation myocardial infarction.
